# Animal Venom in Modern Medicine: A Review of Therapeutic Applications

**DOI:** 10.3390/toxins17080371

**Published:** 2025-07-28

**Authors:** Euikyung Kim, Du Hyeon Hwang, Ramachandran Loganathan Mohan Prakash, Ravi Deva Asirvatham, Hyunkyoung Lee, Yunwi Heo, Al Munawir, Ramin Seyedian, Changkeun Kang

**Affiliations:** 1 College of Veterinary Medicine, Gyeongsang National University, Jinju 52828, Republic of Korea; pooh9922@hanmail.net (D.H.H.); mohanprakash111@gmail.com (R.L.M.P.); devabiochem@gnu.ac.kr (R.D.A.); leehy@gnu.ac.kr (H.L.); yunwi.heo@kitox.re.kr (Y.H.); ckkang@gnu.ac.kr (C.K.); 2 Institute of Animal Medicine, Gyeongsang National University, Jinju 52828, Republic of Korea; 3 Pathology Laboratory, Medical Faculty, University of Jember, Jember 68126, Indonesia; almunawir.fk@unej.ac.id; 4 Department of Pharmacology, Bushehr University of Medical Sciences, Bushehr 14174, Iran; raminseyedian@gmail.com

**Keywords:** animal venom, venom peptides, drug discovery, ion channels, therapeutics, captopril, ziconotide, bioactive compounds

## Abstract

Animal venoms are complex biochemical secretions rich in highly potent and selective bioactive molecules, including peptides, enzymes, and small organic compounds. Once associated primarily with toxicity, these venoms are now recognized as a promising source of therapeutic agents for a wide range of medical conditions. This review provides a comprehensive analysis of the pharmacological potential of venom-derived compounds, highlighting their mechanisms of action, such as ion channel modulation, receptor targeting, and enzyme inhibition. Successful venom-derived drugs like captopril and ziconotide exemplify the translational potential of this biological arsenal. We discuss therapeutic applications in cardiovascular diseases, chronic pain, cancer, thrombosis, and infectious diseases, as well as emerging peptide candidates in clinical development. Technological advancements in omics, structural biology, and synthetic peptide engineering have significantly enhanced the discovery and optimization of venom-based therapeutics. Despite challenges related to stability, immunogenicity, and ecological sustainability, the integration of AI-driven drug discovery and personalized medicine is expected to accelerate progress in this field. By synthesizing current findings and future directions, this review underscores the transformative potential of animal venoms in modern pharmacotherapy and drug development. We also discuss current therapeutic limitations and how venom-derived compounds may address unmet needs in specific disorders.

## 1. Introduction

Animal venoms are sophisticated biochemical arsenals composed of a diverse array of bioactive molecules, including peptides, enzymes, and non-proteinaceous compounds, that have evolved for predation and defense. While these venoms have long been associated with toxicity and lethality, recent decades have seen a growing recognition of their potential as a rich source of pharmacologically active agents [1,2]. Venom-derived compounds are often highly selective and potent, characteristics that are desirable in therapeutic development [3]. The translation of venom components into clinically useful drugs has already yielded notable successes, such as captopril, derived from the venom of the Brazilian pit viper *Bothrops jararaca*, which laid the foundation for modern antihypertensive therapies [4,5].

The modern resurgence of interest in animal venoms is driven not only by the unique pharmacological properties of venom components but also by advancements in omics technologies, peptide synthesis, and structural biology. These tools have enabled researchers to isolate, characterize, and optimize venom-derived compounds with greater precision, facilitating their incorporation into drug development pipelines [6,7]. The diversity of venomous species—ranging from snakes and scorpions to cone snails, spiders, and jellyfish—has further broadened the landscape of potential drug candidates [1,8,9].

Animal venom-derived peptides and proteins exhibit a wide range of biological activities, including analgesic, anticoagulant, antihypertensive, anticancer, antimicrobial, and neuroprotective effects [10,11,12,13]. Their mechanisms of action often involve modulation of specific ion channels, receptors, or enzymes, enabling highly targeted therapeutic interventions [3,14]. As such, venom-based drug discovery represents a promising frontier in biomedical research, particularly for diseases that remain challenging to treat with conventional small molecules [3,10].

This review aims to provide a comprehensive overview of animal venoms as a source of novel therapeutics, with a focus on their biochemical composition, mechanisms of action, and current and emerging applications in modern medicine. By integrating findings from molecular pharmacology, structural biology, and clinical studies, this work highlights the translational potential of venom-derived compounds and the innovative methodologies driving their development.

## 2. Biochemical Composition of Animal Venoms

Animal venoms are complex secretions comprising a heterogeneous mixture of biologically active molecules, including peptides, proteins, enzymes, nucleotides, lipids, and small organic compounds [9,15]. The composition of venom varies significantly across species, reflecting evolutionary adaptations to different ecological niches and prey types [16]. In general, venom components can be broadly categorized into enzymatic and non-enzymatic constituents. Enzymatic components such as phospholipase A2 enzymes (PLA2s), metalloproteinases, serine proteases, hyaluronidases, and L-amino acid oxidases play roles in tissue degradation, hemorrhage, inflammation, and disruption of homeostasis [17,18,19]. Non-enzymatic components, including neurotoxins, cardiotoxins, cytotoxins, and ion channel blockers, typically target specific cellular receptors and channels with remarkable potency and specificity [20,21].

Recent proteomic and transcriptomic studies have revealed even greater complexity within venom compositions, uncovering novel low-abundance components with potential pharmacological activities that were previously overlooked [22]. Such components may include unique disulfide-rich peptides, natriuretic peptides, and bradykinin-potentiating peptides, which expand the therapeutic landscape of venoms for conditions such as hypertension and chronic pain [23].

Snake venoms are among the most extensively studied and serve as prototypical models for venom pharmacology. Elapid venoms (e.g., from cobras and kraits) are predominantly neurotoxic, containing three-finger toxins (3FTxs) and dendrotoxins that block or modulate ion channels and neurotransmitter receptors [24,25]. In contrast, viperid venoms are rich in hemotoxic and cytotoxic components such as snake venom metalloproteinases (SVMPs), serine proteases, and disintegrins, which impair coagulation, damage vasculature, and disrupt extracellular matrix integrity [26,27].

Beyond snakes, cone snails (*Conus* spp.) produce conotoxins—small, disulfide-rich peptides with exquisite specificity for voltage-gated ion channels, nicotinic acetylcholine receptors, and G-protein-coupled receptors. These peptides have become valuable pharmacological tools and therapeutic leads, exemplified by ziconotide, an FDA-approved analgesic for chronic pain management [28,29]. Similarly, arachnid venoms (e.g., from spiders and scorpions) contain neuropeptides that modulate sodium, potassium, and calcium channels, offering potential for treating neurological disorders and pain [3,30].

Marine venoms, such as those from jellyfish and sea anemones, contain unique bioactive peptides and proteins with hemolytic, cytolytic, and neurotoxic properties [31,32]. These venoms are relatively underexplored compared to terrestrial venoms but represent an expanding frontier in venom-based drug discovery [33]. Emerging research suggests that marine-derived toxins may provide novel scaffolds for ion channel modulation and cancer therapy, further broadening the application of venom-derived compounds in modern medicine [23].

Additionally, pore-forming toxins (PFTs), including bee melittin, sticholysins from sea anemones, and potent porins from cubozoan jellyfish, represent a critical component of many venoms [34]. PFTs function by forming transmembrane pores, leading to cell lysis or modulation of cellular ion gradients. While their direct therapeutic application may be limited due to cytotoxicity, PFTs are increasingly being investigated as molecular tools for targeted drug delivery and cancer therapy, and as models for synthetic pore-forming agents.

Overall, the structural and functional diversity of venom components across taxa underscores their immense potential as molecular scaffolds for the development of novel therapeutics [10,30].

## 3. Mechanisms of Action of Venom Components

**Figure 1:**
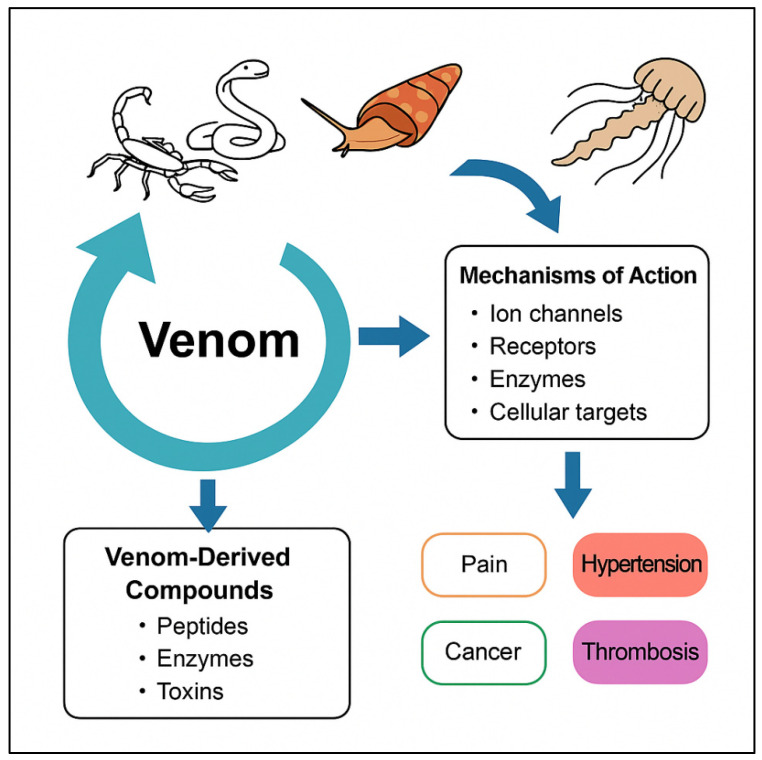
Overview of venom-based drug discovery pipeline.

The biological activity of venom-derived compounds is primarily mediated through their specific interactions with cellular targets, including ion channels, membrane receptors, enzymes, and components of the extracellular matrix. These interactions are often characterized by high affinity and selectivity, enabling venom components to serve as powerful modulators of physiological pathways [35,36] (Figure 1).

Neurotoxins are a prominent class of venom components that target voltage-gated sodium (NaV), potassium (KV), and calcium (CaV) channels. For instance, α-scorpion toxins and sea anemone peptides delay the inactivation of NaV channels, resulting in prolonged neuronal depolarization and paralysis [37,38]. Conversely, spider toxins such as ω-agatoxins and µ-agatoxins can inhibit CaV and NaV channels, disrupting synaptic transmission [39]. These highly specific modulators have been extensively used in neurophysiological research and are being explored as templates for drug development in epilepsy, pain, and cardiac arrhythmias [40,41].

Another well-characterized mechanism is the inhibition of angiotensin-converting enzyme (ACE) by bradykinin-potentiating peptides (BPPs) from viper venoms. BPPs inhibit the conversion of angiotensin I to angiotensin II, thereby reducing vasoconstriction and lowering blood pressure—a mechanism that led to the development of captopril and other ACE inhibitors [42,43]. Similarly, conotoxins from cone snail venom act on nicotinic acetylcholine receptors and N-type CaV channels, offering therapeutic value in neuropathic pain and spasticity [44,45].

The schematic in Figure 1 illustrates the translational pipeline for venom-derived compounds into modern therapeutics. The process includes the following: (i) toxin sourcing; (ii) determination of biological activity (e.g., ion channels, receptors, enzymes); (iii) molecular structure identification (peptides, enzymes, toxins); (iv) toxin production; (v) pharmacodynamic evaluation; and (vi) clinical applications (e.g., pain, hypertension, cancer, thrombosis).

Venom metalloproteinases and serine proteases from viperid snakes degrade components of the extracellular matrix, disrupt basement membranes, and interfere with blood coagulation pathways. These enzymes exert both hemorrhagic and anticoagulant effects, with potential applications in thrombolytic therapy and cancer metastasis inhibition [15,18,26,46]. Disintegrins, another class of venom-derived peptides, bind integrins on cell surfaces and inhibit platelet aggregation, offering antithrombotic potential [47,48]. Disintegrins are a class of low-molecular-weight, cysteine-rich peptides derived from viperid snake venoms. They are characterized by the presence of specific amino acid motifs, such as Arg-Gly-Asp (RGD) or Lys-Gly-Asp (KGD), which enable them to bind selectively to integrins on cell surfaces. By targeting integrins like αIIbβ3 on platelets, disintegrins inhibit fibrinogen binding, thereby preventing platelet aggregation and offering potential as antithrombotic agents. The therapeutic potential of disintegrins has been harnessed in the development of antiplatelet drugs [47]. For instance, tirofiban (Aggrastat) is a nonpeptide glycoprotein IIb/IIIa inhibitor inspired by the RGD motif found in snake venom disintegrins. Tirofiban competes with fibrinogen for binding to the αIIbβ3 integrin, effectively inhibiting platelet aggregation and reducing the risk of thrombotic cardiovascular events.

Ion channel modulation is also a key mechanism underlying analgesic and neuroprotective activities. For example, the conotoxin MVIIA (ziconotide) selectively blocks N-type CaV channels, reducing neurotransmitter release and thereby alleviating chronic pain [45,49]. Likewise, spider venom peptides that target acid-sensing ion channels (ASICs) and transient receptor potential (TRP) channels are under investigation for treating pain and inflammatory conditions [50,51].

Overall, the specificity and potency of venom components in modulating key physiological pathways underscore their therapeutic relevance. By acting on defined molecular targets, venom-derived molecules provide invaluable templates for drug discovery and a deeper understanding of pathophysiological mechanisms [52].

## 4. Therapeutic Applications of Venom-Derived Compounds

Venom-derived compounds have shown substantial promise in the development of novel therapeutics, owing to their unique ability to modulate specific molecular targets with high affinity and selectivity [53]. Several peptide- and protein-based drugs originating from animal venoms have been approved for clinical use, while many others are in various stages of preclinical or clinical development (Table 1) [30,54,55]. These therapeutics span a broad range of medical applications, including cardiovascular diseases, chronic pain, diabetes, and cancer.

One of the most successful examples is captopril, the first angiotensin-converting enzyme (ACE) inhibitor approved for the treatment of hypertension and heart failure. It was developed based on bradykinin-potentiating peptides derived from the venom of *Bothrops jararaca* [4,56]. This breakthrough not only validated venom-derived peptides as drug candidates but also opened the door for further exploration of cardiovascular applications. Other notable ACE inhibitors, such as enalapril and lisinopril, were developed as synthetic analogs of captopril, demonstrating how venom-based scaffolds can serve as starting points for rational drug design [57,58].

In the area of pain management, ziconotide (Prialt^®^) represents a landmark achievement. This synthetic analog of ω-conotoxin MVIIA, derived from Conus magus, selectively inhibits N-type calcium channels and is approved for the treatment of severe chronic pain in patients who are refractory to opioids [29,49]. Unlike opioids, ziconotide does not act via opioid receptors and is not associated with respiratory depression or dependence, making it a valuable alternative in pain pharmacotherapy [59,60].

Venoms have also contributed to the development of antiplatelet agents. Tirofiban and eptifibatide, which are based on disintegrins found in viper venoms, act as glycoprotein IIb/IIIa inhibitors that prevent platelet aggregation, and are used to manage acute coronary syndromes and during percutaneous coronary interventions [61,62]. These agents exemplify how venom proteins that naturally interfere with hemostasis can be adapted for cardiovascular therapeutics.

Exenatide (Byetta^®^), a synthetic form of exendin-4 originally derived from the venom of the Gila monster (Heloderma suspectum), is a GLP-1 receptor agonist approved for the treatment of type 2 diabetes mellitus. It improves glycemic control by enhancing glucose-dependent insulin secretion, suppressing inappropriate glucagon release, and delaying gastric emptying. Additionally, exenatide has been shown to promote modest weight loss due to its appetite-suppressing effects, contributing to its clinical utility in patients with type 2 diabetes who are overweight or obese [63]. Tirzepatide (Mounjaro™) is a novel synthetic dual agonist of the GLP-1 and glucose-dependent insulinotropic polypeptide (GIP) receptors, often referred to as a “twincretin.” It has been recently approved for the treatment of type 2 diabetes and has demonstrated significant efficacy in improving glycemic control while inducing substantial weight loss in clinical trials. Tirzepatide enhances glucose-dependent insulin secretion, reduces glucagon levels, delays gastric emptying, and improves insulin sensitivity, making it a promising therapeutic option for managing both hyperglycemia and obesity in patients with type 2 diabetes. These venom-derived and venom-inspired therapeutic agents highlight the translational potential of peptide toxins and their analogs in addressing metabolic disorders, demonstrating how bioactive components from animal venoms can inform the development of effective treatments for chronic diseases [64].

Beyond cardiovascular and pain indications, venom-derived peptides are being actively investigated for oncological applications. For instance, chlorotoxin, a peptide from scorpion venom, has demonstrated the ability to bind selectively to matrix metalloproteinase-2 (MMP-2), a protein overexpressed in glioma cells. This specificity has enabled its use in tumor imaging and as a delivery vehicle for targeted therapies [65,66,67]. Likewise, melittin, the principal peptide in bee venom, exhibits anticancer properties through induction of apoptosis, inhibition of angiogenesis, and disruption of cancer cell membranes, although its non-specific cytotoxicity remains a limitation [68,69]. Additionally, melittin has been evaluated for pain management in rheumatoid arthritis, with clinical studies indicating reductions in pain and inflammation [70]. In fact, bee venom has long been used for this purpose in Oriental medicine, particularly in Korean traditional medicine as a form of pharmacopuncture called bee venom acupuncture (BVA).

Recent studies have also explored the antimicrobial and antiparasitic potential of venom peptides. Certain defensin-like peptides from spider and scorpion venoms exhibit broad-spectrum antibacterial and antifungal activity, possibly by disrupting microbial membranes [71,72]. Additionally, peptides from cone snail and wasp venoms have demonstrated inhibitory effects against protozoan parasites such as Plasmodium and Trypanosoma, suggesting utility in tropical infectious diseases [73,74].

The therapeutic versatility of venom-derived compounds is being expanded further through synthetic biology and peptidomimetic design, enabling the enhancement of their pharmacokinetic properties and specificity [30,75]. These efforts are critical for overcoming challenges related to peptide stability, immunogenicity, and delivery, thereby increasing the clinical viability of venom-based drug candidates. These examples underscore the translational potential of venom-derived compounds from bench to bedside, highlighting their impact in addressing conditions such as chronic pain, hypertension, diabetes, cancer pain, and inflammatory disorders.

**Table 1:** Peptidomimetic venom drugs and candidates in clinical use or development.

Drug Name	Source Toxin	Origin Animal	Target/Mechanism	Indication	Clinical Status	Refs.
Captopril	Bradykinin-potentiating peptide	*Bothrops jararaca* (Viper)	ACE inhibitor	Hypertension, heart failure	FDA-Approved	[4,56,57,58,76]
Ziconotide (Prialt^®^)	ω-Conotoxin MVIIA	*Conus magus* (Cone snail)	N-type Ca^2+^ channel blocker	Chronic neuropathic pain	FDA-Approved	[45,59,60]
Eptifibatide (Integrilin^®^)	Barbourin analog (Disintegrin)	*Sistrurus miliarius* (Viper)	GPIIb/IIIa inhibitor	ACS, PCI	FDA-Approved	[61,62]
Tirofiban (Aggrastat^®^)	Echistatin analog	*Echis carinatus* (Saw-scaled viper)	GPIIb/IIIa antagonist	Coronary artery disease	FDA-Approved	[77]
Exenatide (Byetta^®^)	Exendin-4	*Heloderma suspectum* (Gila monster)	GLP-1 receptor Agonist	Type 2 diabetes, weight loss	FDA-Approved (tirzepatide)	[63,64]
Desmoteplase	DSPA α1	*Desmodus rotundus* (Vampire bat)	Clot-specific plasminogen activator	Ischemic stroke	Completed Phase II	[78]
Chlorotoxin-based imaging agents (e.g., BLZ-100)	Chlorotoxin	*Leiurus quinquestriatus* (Scorpion)	MMP-2 binding	Glioma imaging	Phase I/II	[65,66,67]
Dalazatide (ShK-186)	ShK toxin	*Stichodactyla helianthus* (Sea anemone)	Kv1.3 channel blocker	Psoriasis, MS	Completed Phase I	[79]
Heparin mimetics (e.g., Ancrod)	Ancrod	*Calloselasma rhodostoma* (Pit viper)	Defibrinogenating agent	Stroke, thrombosis	Withdrawn after Phase III	[80]
Huwentoxin-IV analogs	Huwentoxin-IV	*Ornithoctonus huwena* (Spider)	Nav1.7 sodium channel blocker	Analgesia	Preclinical	[81]
D-Melittin–nanoparticle conjugates	Melittin	*Apis mellifera* (Bee)	Membrane disruption	Cancer	Preclinical	[68,82]
Apimol/Apitoxin	Bee venom extract containing melittin	*Apis mellifera* (Bee)	Anti-inflammatory and analgesic pathway	Rheumatoid arthritis pain	Phase II (clinical trials, bee venom extract containing melittin)	[70]

## 5. Technological Advances Facilitating Venom-Based Drug Discovery

The successful translation of venom components into therapeutic agents has been significantly accelerated by recent technological advancements across various scientific disciplines. Innovations in high-throughput screening, transcriptomics, proteomics, and structural biology have enabled comprehensive profiling of venom composition and function, thereby transforming venom research from a descriptive to a mechanistic science [7,83,84].

High-throughput sequencing technologies, particularly next-generation sequencing (NGS), have facilitated the generation of venom gland transcriptomes from a wide array of venomous species. For example, a recent study on *Bothrops asper* and *Bothrops jararaca* performed de novo transcriptome assembly and bioinformatic analysis [85], identifying not only highly expressed toxin families (e.g., SVMPs, SVSPs, PLA2s, CTLs) but also novel low-abundance proteins such as arylsulfatases and glycerophosphodiester phosphodiesterases, which may contribute to venom variability and present opportunities for antivenom development and drug discovery. This approach has allowed for the rapid identification of novel toxin-coding genes and isoforms, greatly expanding the catalog of potential bioactive peptides [86,87]. Complementary proteomic methods such as liquid chromatography–mass spectrometry (LC-MS/MS) provide detailed information on the molecular weight, sequence, and post-translational modifications of venom proteins, enabling the characterization of mature venom components with functional relevance [84].

Advances in bioinformatics and molecular modeling have also played a pivotal role in venom-based drug discovery. In silico techniques such as molecular docking, molecular dynamics (MD) simulations, and quantitative structure–activity relationship (QSAR) modeling are routinely employed to predict binding affinities, optimize pharmacophores, and identify lead compounds prior to synthesis and in vitro validation [88,89,90]. These tools not only reduce the cost and time associated with experimental assays but also provide mechanistic insights that inform rational drug design. For example, a recent pharmacoinformatic study on plant-derived phytochemicals identified potential inhibitors of the venom metalloproteinase Atrolysin by employing high-throughput virtual screening, molecular docking, MD simulations, and density functional theory (DFT) calculations [83]. This integrative approach enabled the identification of compounds with strong binding affinities (−8.7 to −10.2 kcal/mol) and stable dynamic interactions with the target, confirmed by low RMSD values and consistent hydrogen bond formations throughout 100 ns MD simulations. The study further employed DFT calculations to analyze HOMO-LUMO energy gaps, indicating favorable electronic properties for protein interactions, and MMPBSA analysis to validate binding energies, providing a robust pipeline that can guide venom-targeted drug development while reducing the need for early-stage experimental screening.

Synthetic biology and peptide engineering technologies have further enhanced the therapeutic potential of venom-derived compounds. Through recombinant expression systems, including bacterial, yeast, insect, and mammalian cells, researchers can produce venom peptides in large quantities with defined structural fidelity [91]. Chemical synthesis and cyclization strategies allow for the generation of peptide analogs with improved stability, bioavailability, and target selectivity. Moreover, site-directed mutagenesis and incorporation of non-natural amino acids have facilitated structure–activity relationship studies and the development of peptidomimetics with desirable pharmacokinetic properties [92,93].

Structural biology techniques, including X-ray crystallography, cryo-electron microscopy (cryo-EM), and nuclear magnetic resonance (NMR) spectroscopy, have provided atomic-level insights into toxin–target interactions. These approaches enable the elucidation of binding modes and conformational dynamics, which are critical for the rational design of therapeutic analogs [94,95]. Coupled with data from functional assays and pharmacological profiling, these technologies collectively support the identification and optimization of venom-derived drug candidates.

The integration of these advanced methodologies has ushered in a new era of venom research, allowing for systematic and targeted exploration of the vast chemical diversity encoded within animal venoms. As a result, the discovery and development of venom-based therapeutics are becoming increasingly efficient, scalable, and precise, paving the way for next-generation biologics and peptide drugs [96,97].

## 6. Venom-Derived Therapeutics for Unmet Medical Needs

Although there have been significant advancements in modern therapeutics, current treatment options for conditions such as chronic pain, hypertension, and certain cancers often face limitations, including insufficient efficacy, development of resistance, and adverse side effects that reduce patient compliance [98]. For instance, opioid-based analgesics, while effective, are associated with the risk of dependence and tolerance, while conventional antihypertensives may be inadequate in refractory cases [99]. In this context, venom-derived compounds offer unique advantages, such as high potency at low doses, novel mechanisms of action targeting specific ion channels and receptors, and the potential to overcome limitations associated with current therapeutic agents [30]. Thus, exploring animal venoms as a source for developing new therapeutics can address unmet medical needs in specific disorders where current treatments are suboptimal (Table 2).

## 7. Challenges and Future Directions

Despite the promising therapeutic potential of venom-derived compounds, several challenges hinder their seamless translation into clinically approved drugs. One of the foremost obstacles is the complexity and variability of venom composition. Venoms are often composed of hundreds of peptides and proteins, the expression profiles of which can vary significantly depending on species, age, diet, geographical distribution, and even individual physiological states [100,101]. This complexity not only complicates the standardization of venom-derived products but also presents difficulties in reproducibility and scalability for pharmaceutical development.

A related issue is the difficulty in isolating and characterizing low-abundance bioactive components from natural venoms. Even with advanced omics tools, distinguishing pharmacologically relevant molecules from a background of enzymatic or toxic proteins remains a substantial task. Additionally, venom peptides often suffer from poor stability, a short half-life, and low oral bioavailability, limiting their practical application unless chemically modified or delivered via advanced drug delivery systems [30,102].

The issue of immunogenicity is also non-negligible. As exogenous proteins and peptides, venom components can elicit unwanted immune responses, including hypersensitivity and anaphylaxis, particularly upon repeated administration [103,104]. Strategies such as pegylation, cyclization, and nanoparticle encapsulation are being explored to mitigate such risks, but these modifications may alter pharmacodynamics and require extensive validation [105].

Furthermore, the ethical and ecological considerations associated with venom harvesting pose additional constraints. Many venomous species are endangered or difficult to culture in captivity, raising concerns about sustainability and biodiversity loss [106]. Recombinant expression systems and synthetic biology approaches offer alternatives, but they require significant optimization to yield functionally active peptides that mimic natural counterparts in structure and activity [107].

From a regulatory perspective, venom-derived compounds often fall into a gray zone between biologics and small molecules, complicating approval processes. The need for rigorous preclinical and clinical studies, combined with high production costs and uncertain intellectual property pathways, may deter commercial investment despite scientific promise [8].

Looking ahead, future directions in venom-based drug discovery will likely be shaped by the integration of artificial intelligence (AI), machine learning, and systems biology. These approaches can accelerate candidate identification, predict toxicity, and guide structural optimization by mining large-scale datasets of venom sequences, structures, and activities [108]. Moreover, personalized medicine frameworks may allow for the tailoring of venom-based therapies to individual genetic and physiological profiles, particularly in complex diseases such as cancer or autoimmune disorders [109].

Collaborative efforts that bridge academic research, biotechnology, and clinical practice will be essential in advancing venom-derived compounds from bench to bedside. The continued expansion of venom databases, combined with robust validation platforms and translational infrastructure, will determine the success of this promising but still emerging therapeutic frontier.

## 8. Conclusions

Animal venoms represent a vast and largely untapped resource for the discovery of novel therapeutic agents. With their remarkable biochemical diversity and target specificity, venom-derived peptides and proteins have already led to the development of clinically important drugs such as captopril, ziconotide, and tirofiban. Recent advancements in omics technologies, bioinformatics, structural biology, and synthetic biology have transformed venom research into a systematic and translationally oriented field. Despite ongoing challenges related to bioavailability, immunogenicity, and ecological sustainability, continuous innovation in delivery systems and peptide engineering holds promise for overcoming these barriers. Looking forward, the integration of artificial intelligence and personalized medicine strategies may further unlock the therapeutic potential of venom-based compounds. As the scientific community continues to explore and harness this biological arsenal, venom-based drug discovery is poised to contribute significantly to the future of precision medicine. Future research on venom-based therapeutics should focus on addressing the limitations of existing treatments by leveraging the unique mechanisms and high specificity of venom-derived compounds for specific clinical applications.

## Figures and Tables

**Table 2:** Limitations of current therapies and potential advantages of venom-derived therapeutics.

Therapeutic Area	Limitations of Current Treatments	Advantages of Venom-Derived Therapeutics
Chronic Pain	Opioid tolerance, dependence, limited efficacy in neuropathic pain	Novel ion channel blockers (e.g., ziconotide) with high specificity
Hypertension	Refractory cases, side effects with polytherapy	ACE inhibitors derived from snake venom (e.g., captopril)
Cancer Pain	Limited efficacy, opioid-related issues	Crotoxin showing analgesic and potential antitumor effects
Diabetes	Suboptimal glycemic control, hypoglycemia risk	GLP-1 receptor agonists from Gila monster venom (e.g., exenatide)

## Data Availability

Not applicable.
